# Categories of the Patient-Specific Functional Scale Activities in Chronic Neck Pain and Their Relationship to the Neck Disability Index

**DOI:** 10.1155/2024/3126892

**Published:** 2024-09-23

**Authors:** Barbara Van Gorp, Joseph Lesnak, Timothy Fleagle, Kyle Hulshizer, Ashley Nielsen-Wise, Lisabeth Kestel, Carol Vance, Kathleen A. Sluka

**Affiliations:** ^1^ Department of Rehabilitation Therapies University of Iowa Hospitals and Clinics, 200 Hawkins Dr, Iowa City, Iowa 52242, USA; ^2^ Department of Physical Therapy and Rehabilitation Sciences University of Iowa, 500 Newton Rd, 1-252 MEB, Iowa City, Iowa 52242, USA

**Keywords:** functional activities, NDI, neck pain, outcome measures, PSFS

## Abstract

**Intoduction:** Common outcome measures for chronic neck pain are the Patient-Specific Functional Scale (PSFS) and the neck disability index (NDI). The primary aim was to categorize the top-rated, patient-selected functional activity limitations of the PSFS to determine if there were consistent limited functional activities for individuals with chronic neck pain and how these compared to the constructs of activities on the NDI. The secondary aim was to determine the relationship between scores for individuals who completed both the NDI and PSFS.

**Design:** A retrospective review of data extracted from the electronic medical record, EPIC, within two hospital-based outpatient physical therapy clinics within a health care system.

**Methods:** Retrospective analysis was performed on individual's characteristics, self-selected functional activity limitations, and total scores of the PSFS and NDI. Most common categories of self-selected functional activity limitations were developed by practicing physical therapists. These functional activity limitation categories of the PSFS were compared to the activities of the NDI. Mean PSFS total scores were correlated with the NDI total scores with Spearman's test.

**Results:** Participants were individuals with chronic neck pain from January 2013–September 2018 (*n* = 2283). Movement-based activities accounted for 60.8% of the functional activity limitations of the PSFS with the top functional activity limitations being cervical motion and exercise (32%). The PSFS total score moderately correlated with NDI (*r* = −0.50, *p* = <0.01) which may relate to the differences in constructs of the NDI and the top patient-selected PSFS functional activity limitations found in this analysis.

**Conclusion:** The results suggest that individuals with chronic neck pain present with similar categories of self-selected functional activity limitations that differ from activities of the NDI. Additional research is needed to improve outcome measures to capture patient-selected functional activity limitations and an individual's pain experience.

**Summary**

• Common categories of functional activity limitations within the PSFS are present in a large sample of chronic neck pain individuals with the top categories relating to movement.
• PSFS and NDI scores have a moderate correlation which may relate to constructs of the NDI and PSFS.
• Development of a modified NDI or PSFS outcome measure may encourage higher usage, improve reimbursement, and help practitioners develop a therapy plan that includes the patient-generated concerns to capture the patient's pain experience.

## 1. Introduction

Chronic neck pain is a costly and common musculoskeletal disorder in the adult population of the US at approximately 15%[1], yet ranks as high as 70% occurrence at some point during a life span [2]. Chronic neck pain has been associated with increased health care costs and high disability [3]. Disability and decreased function are common measures used to determine one's ability to return to work [4]. In 2016, Americans spent $134.5 billion on lower back pain and neck pain. When compared to 1996, this increase was mostly related to an increase in outpatient care. Yet there is difficulty in concluding if this spending has led to better outcomes for the management of chronic neck and lower back pain [5].

The rate of physical therapy reimbursement is commonly associated with changes in functional outcome tests and measures completed by both the individual and the physical therapist. The burden of outcome measure assessment and data collection for physical therapists continues to increase and is related to reimbursement. There is a progression towards shifting pay from volume-based traditional fee-for-service to value-based payment models using outcome measures by both the Centers for Medicare and Medicaid Services (CMS) and private insurers. [6, 7] (CMS and APTA.org) Currently, there is no requirement by CMS within the United States for the use of specific outcome measure tests in outpatient physical therapy. However, various organizations are beginning to recommend the Patient-Specific Functional Scale (PSFS) as a core outcome measure [8].

There are a variety of outcome measures used in physical therapy clinics to assist in the assessment of function, disability, and goals. Two commonly used outcome measures in physical therapy practice for individuals with neck pain are the PSFS and the neck disability index (NDI). The NDI, developed in 1991 [9], is a revised version of the Oswestry Disability Index (ODI) for low back pain [10, 11] that is specific to neck pain and examines 10 pre-selected items that encompass a broad range of domains including concentration, pain, a variety of activities, work, sleep, and headaches. The PSFS, developed in 1998, is a self-report measure that examines functional activity limitations by having individuals self-identify what activity they would like to do better and rating their difficulty in performing this activity and is, thus, a unique patient-centered measure [12–14]. Several groups recommend or require the use of the PSFS for musculoskeletal pain assessment, including neck pain. This includes Dutch clinical practice guidelines in primary care clinics, the Workplace Safety and Insurance Board of Ontario, Canada, and the New Zealand National Accident Compensation Corporation [8, 15, 16]. As clinicians aim to assess patients' function accurately and provide individualized care, it is important to understand functional limitations and how these relate to disability.

The primary purpose of this study was to retrospectively determine the most frequently reported patient self-selected functional activity limitations from the PSFS in individuals with chronic neck pain and group the activities into similar categories. A secondary aim was looking at the relationship between the NDI, and the top functional activity limitations of the PSFS, a self-reported measure, and to determine if the scores give a similar understanding of the individuals' current experience. We hypothesized that the most frequently reported functional activity limitations from the PSFS will be uniquely different from the NDI.

## 2. Methods

This study was a retrospective pragmatic chart review of physical therapy visit documentation in the EPIC system with the primary purpose of determining the most frequently reported PSFS functional activity limitations. This study was approved by the Institutional Review Board at the University of Iowa. Data from initial evaluations performed in the University of Iowa Hospital and Clinics outpatient physical therapy clinics was pulled from patient records with the diagnosis of chronic neck pain based on the ICD-10 diagnostic codes (Table 1) within the EPIC system. Only those with a duration of 3 months or longer were included. Inclusion criteria were individuals between the ages of 18–92 years, a PSFS score present at the initial evaluation, and individuals seen for an initial outpatient evaluation in either of the two physical therapy hospital-based outpatient clinics of the University of Iowa Health Care (UIHC) system from January 2013–September 2018 (*n* = 2283). Individuals were not included if they were given an acute neck pain diagnostic ICD-10 code or if the episode of care was billed as an inpatient or from the short-stay outpatient unit. Health Care Information Systems (HCIS) extracted data from the EMR of the institution (EPIC system). Identifiable characteristics such as name and patient medical record numbers were removed by HCIS staff, and individuals were given an arbitrary number to maintain confidentiality. Data extracted by the HCIS team included the following: sex, age, date of onset of symptoms, date of initial evaluation, PSFS scores, PSFS functional activity limitations listed by the individual, and NDI scores.

The three-item PSFS is utilized as one of the primary patient-reported outcome measures within the UIHC Rehabilitation Department and incorporated within the EMR. This three-item PSFS utilizing a standardized script (Supporting Information Appendix 1) is the only required patient-reported outcome measure to be completed within the physical therapy assessment and documented in the electronic chart. Scoring ranges from 0 to 10, where patients rate their ability to complete an activity on an 11-point scale based on a level prior to an injury or problem where 0 is unable to complete and 10 is able to complete as prior to this injury or problem [14]. The NDI (Supporting Information Appendix 2) is an option within the UIHC system, based on the clinical reasoning of each outpatient physical therapist but is not required. There are 10 questions with each question being scored on a 6-point scale, where 0 is no disability to 5 being complete disability. All 10 sections are then totaled. Scoring ranges from 0 to 50, with 0 being the best score and 50 the worst. The score also can be converted to a percentage (0%–100%)[9].

The NDI has been considered the gold standard outcome measure for examining disability in individuals with neck pain and has been shown to have test-retest reliability and construct validity in previous research [9–11]. The PSFS has high construct validity and test-retest reliability and responsiveness to change in individuals with cervical pain [17, 18]. Despite this, previous studies show no difference in the ability to detect change using the PSFS when compared to the NDI [14], and some question the validity and reliability of the PSFS for chronic neck pain [19, 20]. There is low-quality evidence of concurrent associations between the PSFS and the NDI suggesting these may be examining different constructs for patients with chronic neck pain and cervical radiculopathy [12, 21]. Other studies have reported that the NDI has had limited assessment of content validity [10, 22, 23].

The study team consisted of four licensed physical therapists who all have more than 20 years of clinical and/or research experience from both UIHC and the University of Iowa Physical Therapy and Rehabilitation Science Department within the Carver College of Medicine. The team also included a physical therapy PhD student and a Doctor of Physical Therapy student in their final year.

Chronic neck pain inclusion criteria were confirmed by calculating the difference between the date of onset of symptoms and the date of initial evaluation. A time frame greater than 3 months was defined as chronic neck pain [2]. Individuals were included only if a PSFS score was present for the initial evaluation (*n* = 2283) (Supporting Information Appendix 3).

Each of the functional activity limitations from the 2283 individuals listed in the PSFS was grouped into similar movement and positional categories based on the description listed in the functional activity limitation entry within the EMR. Categories of the PSFS functional activities limitations were initially determined by a practicing physical therapist (> 20 years experience) in a preliminary dataset consisting of 3 months of data extracted by HCIS from the EMR. The resulting categories from this preliminary dataset were then reviewed by three other physical therapists and finally categorized into 17 unique categories. These 17 categories were then reviewed by physical therapy students to determine if any of the categories were similar. Three of the 17 categories were determined to have similar actions (sitting, reading, and studying) and were combined into a single category for a final set of 15 categories to be used for the full analysis.

For categorizing each of the PSFS functional activity limitations, each functional activity limitation was categorized by two independent investigators into one of the 15 categories. A total of 4260 functional activity limitations from the PSFS were initially reported from 2283 individuals to be sorted into the common categories. A Cronbach alpha coefficient of internal consistency was calculated for participants who gave 2 or 3 functional activity limitations. Investigators were trained in sorting into categories and preliminary results of sorting from a small subset were confirmed for each pair of investigators. A third independent investigator reconciled undifferentiated categories from the two independent investigators for a total of 333 functional activity limitations needing reconciliation. While sorting into common categories, 33% of the subjects at the initial evaluation had incomplete or inappropriate data entries and were excluded, resulting in the removal of 1651 of the functional activity limitations listed in the PSFS data analysis. This resulted in a total of 2609 functional activity limitations from the PSFS for analysis of activity description. The percentages of each of the 15 categories were compared to the total activities reported for all subjects and sorted by sex. Results were qualitative and descriptively compared to the NDI categories.

A subset of 551 individuals included both the NDI and PSFS total scores at the initial evaluation. This subset of individuals with functional activity limitations that included NDI scores and PSFS total scores was analyzed for categorical percentages to determine if there were similar categories of functional activity limitations in this smaller subset. The percentages of each of the 15 categories in this subset were compared to the total functional activity limitations data. A correlation analysis was completed to determine the direction and strength of the linear relationship between the subset of PSFS mean total scores and NDI total scores in this subset. The PSFS and NDI were not normally distributed as tested by the Shapiro–Wilk test (R-program). Therefore, we performed a Spearman correlation between the total scores for the NDI and the PSFS. Spearman's correlation was determined to be utilized as we were analyzing ordinal values for two variables.

## 3. Results

A total of 2283 individuals were included for analysis of the PSFS entries of functional activity limitations. The EPIC system EMR within the UIHC system is designed with controls that limit errors in the entry for total scoring of the PSFS, eliminating any values that would be outside the scoring range (0–10) for the activities entered at each individual functional activity limitation. A total of 4260 functional activity limitations from the PSFS were initially reported before removing incorrect responses. Utilizing the 3-item form of the PSFS, 2283 individuals self-reported at least 1 functional activity limitation on the PSFS, 1218 individuals self-reported 2 activities and 759 individuals had 3 functional activity limitations in the PSFS. Calculations of the Cronbach alpha coefficients were completed for individuals with 3 functional activity limitations scores (alpha = 0.75) and those individuals with 2 functional activity limitations scores (alpha = 0.74). Both sets of results indicated acceptable levels of internal consistency among the scores.

**Figure 1:**
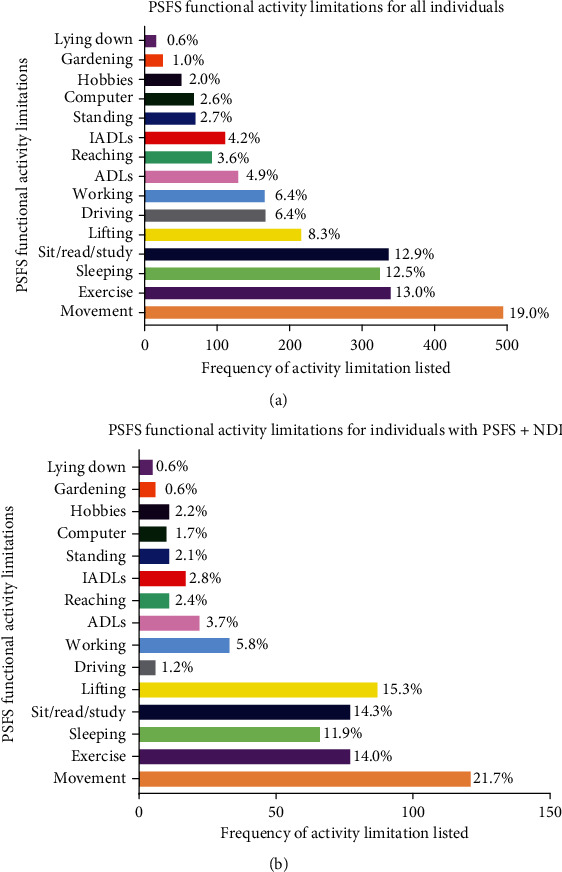
(a) Bar graph representing totals in percent per category of functional activity limitations on the PSFS for all subjects with PSFS scores at initial evaluations. IADLS, instrumental activities of daily living; ADLs, activities of daily living. (b) Bar graph representing totals in percent per category of functional activity limits for subjects with both NDI and PSFS scores at initial evaluation. IADLS, instrumental activities of daily living; ADLs, activities of daily living.

The age of patients ranged from 18 to 92 years old. Of these patients 65% were female. The average length from the onset of time until the initial evaluation was 31.63 ± 48.9 months (mean ± SD) for women, and 27.85 ± 41.2 was for men. The mean PSFS score at initial evaluation was 4.81 ± 2.25 (mean ± SD). Similar distributions of functional activity limitations were observed for males and females. We reported all data with males and females pooled together. The top two rated functional activity limitations for the PSFS were movement-related, defined as cervical or head movement (*M* = 19%), and exercise (*E* = 13%) which accounted for approximately 32% of responses (Table 2, Figure 1(a)). Functional activity limitations from the PSFS that had movement-related qualities included activities of daily living (ADLs), exercise, lifting/carrying, reaching, gardening, hobbies, work, and neck-specific movements accounted for 60.8% of the functional activity limitations reported.

Using the subset of 551 individuals that had both PSFS and corresponding NDI total scores at initial evaluation, categorical comparisons were performed. This subset distribution of PSFS functional activity limitations is similar to that found for all individuals (Figure 1(a), 1(b)). The top three rated functional activity limitations of the PSFS in this subset were the same as the full sample (cervical and head movement (*M* = 22%, lifting (*L* = 15%) and exercise (*E* = 14%)) and movement accounted for the majority of the limitations (66%) (Figure 1(b)). Figures 1(a) and 1(b) show the similarities of percentages per category for the PSFS functional activity limitations for all individuals and the subset group with both NDI and PSFS total scores.

**Figure 2:**
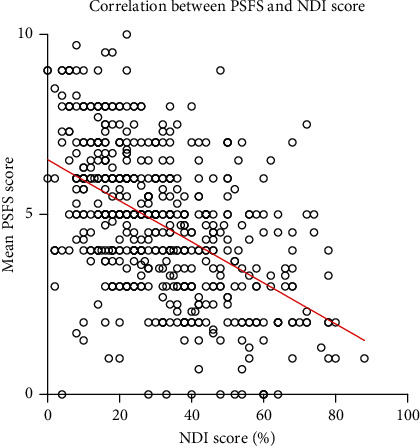
Scatter plot with correlation between the Patient Specific Functional Scale (PSFS) score and neck disability index (NDI) score. (*r* = −0.50, *p* < 0.01).

The NDI movement activities included driving, lifting, and work, which accounted for 22% of the functional activity limitations listed in the PSFS in this subset of the patients. Qualitative comparison between the NDI and functional activity limitations reported on the PSFS showed that functional activity limitations specifically related to cervical movement and exercise were not represented in the NDI, whereas the NDI had categories for headache, pain, and concentration that were not described by patients in the PSFS functional activity limitations. The mean NDI score was 30.9 ± 17.78 (mean ± SD). NDI scores and mean PSFS total scores had a moderate negative correlation (*r* = −0.50 [95%*CI* 0.44 − 0.57], *p* < 0.01) (Figure 2).

## 4. Discussion

### 4.1. Primary Aim

The current study showed that the majority (60.8%) of reported functional activity limitations were related to movement-based functional activities. The other 39.2% of functional activity limitations in the PSFS included sedentary behaviors and a smaller percentage of functional activity limitations that incorporated possible movement associated with upper extremity use. The results of this study are uniquely different from other studies that have reported patient outcomes for the PSFS. One prior study mapped the PSFS functional activity limitations to the International Classification of Function, Disability, and Health (ICF) for a multitude of body functions and structures and reported 80% of functional activity limitations reported by individuals mapped to the activities component within the ICF [24]. The activity component of the ICF defines activities as tasks or actions executed by an individual. Limitations of these activities are defined by the ICF as difficulties the individual has executing these activities. The ICF has different chapters represented under the IFC component of activities and participation. These include communication, mobility, self-care, domestic life, interpersonal interaction and relationships, work and employment, and community and social life. Andelic was found within the activity component, 79.9% related to mobility for all diagnosis. The largest body region represented in the mobility chapter was the neck (29.3%). This supports this current study which shows mobility to be the top category for the PSFS functional activity limitations self-reported in individuals with chronic neck pain.

Another recent study in 2020 reported on a modified PSFS 2.0 [25] with an aim of determining if scores on the original PSFS differed from the modified PSFS 2.0 in individuals with neck pain to determine content validity. De Graaf et al. [25] first had individuals identify functional activity limitations specific to themselves as in the original PSFS and then provided individuals with an example list of functional limitation activities. This included a list of 19 activities which was created using the most mentioned activities by individuals with neck pain according to the neck pain guidelines. The results from 100 individuals with neck pain indicate that individuals prefer the PSFS 2.0 version, but the PSFS original is appropriate. This study was not comparing categories between patient self-reported activity limitations and the example list, but it may be a framework to determine in the future if the categories found in this current study are similar to the example list derived by de Graaf in 2020 to assess content validity.

The PSFS 2.0 has also been compared to the Cervical Radiculopathy Impact Scale (CRIS) subscale 3—activities and action [26]. This study was specific to individuals with cervical radiculopathy and utilized semi-structured interviews to assess individual preferences. From the 22 individuals included in this study, the most frequently reported functional limitation was overhead activities for the PSFS 2.0. In addition, 82% of individuals preferred the PSFS 2.0 to present their specific functional limitations over the CRIS subscale 3, which has 6 predetermined activities. The current study categorized PSFS functional activity limits of 2283 individuals to determine categories of self-reported functional activity limitations of the PSFS by individuals and showed movement-related categories were the most common. This is consistent with Thoomes et al. [26] who found overhead activities were the most common reported functional activity limitation in the PSFS 2.0 for individuals with cervical radiculopathy. Understanding the most frequent self-reported functional activity limitations from the PSFS in a large sample size may give added value for example lists for the PSFS 2.0 specific to chronic neck pain, considering the difficult and lengthy process of developing new patient-reported outcome measures. Categories derived directly from patient self-report that will help to guide treatment in an individualized manner. Further comparison of the top categories of the functional activity limitations of individuals with neck pain to the ICF chapters and to the Neck Pain Guidelines may be beneficial.

### 4.2. Secondary Aim

The secondary aim looked at the relationship between the PSFS and NDI scores in a smaller subset of individuals (551) where both scores were present at the initial evaluation. Qualitative analysis of the PSFS in comparison to the NDI showed that functional activity limitations related to movement were not fully represented in the NDI and thus there was only a moderate correlation between the NDI and the PSFS scores. The NDI includes driving, lifting, and work which overlapped with categories of functional activity limitations on the PSFS. Other sections of the NDI include pain intensity, concentration, and headaches which were not accounted for in the PSFS showing that the NDI evaluates constructs beyond functional activity limitations. A recent study examined the content validity of NDI where individuals with neck pain ranked the relevance and comprehensiveness of NDI on an 11-point scale and clinician experts provided feedback on domains [22]. Four out of 10 individuals with neck pain thought all items were relevant with the remaining 6 individuals stating most of the items of the NDI—personal care, lifting, work, sleeping, and driving, were not relevant to them. These individuals thought items missing from the NDI included computer work, sports, and mobility. This study by Ailliet reinforces the results of our current study which demonstrate the top categories of functional activity limitations on the PSFS relate to movement and that the PSFS can examine these missing items in a patient-specific manner.

The PSFS scores in this analysis suggested moderate limitations in function [13, 19] while the NDI scores suggested severe disability [9, 10]. These differences in instrument interpretation of outcome measures complicate factors for the clinician on treatment decisions and could influence payment models. For example, individuals with higher disability ratings result in greater physical therapy reimbursement during evaluation [27]. Further, the treatment of an individual with a severe disability could lead to a longer episode of care, poor outcomes, and clinician bias [28, 29]. In individuals with chronic neck pain, those with a higher ranking of disability are more likely to struggle to improve within a reasonable time frame or to not return to work and live with higher years of disability [6, 29, 30]. Loss of function and decreased quality of life for individuals with neck pain is a significant societal burden resulting in significant disability [7, 31]. In fact, disability payments for neck pain are second only to low back pain in the United States [32, 33]. These differences in disability and functional activity limitations suggest different constructs are measured, but are often misinterpreted by payors and at times clinicians.

Clinicians are continually encouraged to improve efficiency and increase productivity. The use of patient-reported outcome measures can improve efficiency for evaluations and treatment plans. Interestingly, a recent study found that few outcome measures besides the numeric pain rating or Visual Analog Scale (VAS) were routinely used in clinical practice to assess individuals with neck pain [20] but concluded that additional outcomes were needed. Most clinicians suggested the reasons for the use of outcome measures were to fulfill documentation requirements (78%), for setting treatment goals (41%), and communicating with individuals undergoing treatment (40%).

The PSFS is not considered a disability measure but should rather be considered for developing patient-specific treatment plans to provide a breadth of activity limitations that are not assessed with the NDI. Thus, as practice evidence continues to evolve, the development of a modified NDI or additional assessment of a PSFS with input based on multiple reviews of the self-reported functional activity limitation categories for chronic neck pain may ease clinician burden and improve adherence to outcome measure assessment in the daily routine of physical therapy evaluations.

### 4.3. Limitations

This retrospective, pragmatic qualitative study included a large sample size examining functional limitations from individuals with chronic neck pain presenting to physical therapy. These functional limitations may not represent all individuals with neck pain that present to other clinicians such as primary care. Exclusion criteria were based on age, ICD-10 diagnostic codes, and initial evaluation completion of outcome measures. Of note, we excluded approximately 33% of the total entries for the PSFS due to error in entry by clinicians. Instead of stating just a functional activity limitation given by an individual, clinicians would change this to a short- or long-term goal. Other errors related to not including a score associated with the activity. While we did assess the PSFS for internal consistency, caution should be used on interpretation because the scale is not based on a reflective model [8]. There also may have been an inherited selection bias in this search based on the inclusion of a broad definition of chronic neck pain as defined by ICD-10 diagnostic codes. This study included data from 2013 to 2018 from two hospital-based outpatient clinics within the same health care system and, thus, may not reflect that observed in all outpatient clinics. The PSFS was administered by practicing clinicians as part of daily practice, a standardized script is given to each clinician. However, it is likely that all clinicians did not follow the script as approximately 33% of the total entries for the PSFS due to errors in entry by clinicians were excluded. On the other hand, the use of a large sample size with collection over a 5-year period from a large physical therapy setting was a strength of the study. Finally, while data analysis included four practicing physical therapists, one PhD student physical therapist, and DPT student physical therapists in their final year, reliability between investigators was not assessed numerically. However, discrepancies in category selection were decided by practicing physical therapists with each over 20 years experience.

## 5. Conclusion

Clinically, the use of multiple outcome measures is limited due to time and the focus on productivity standards. Ease of use and documentation ranks high for practitioners as Jette found only 48% of the participants used outcome measures with length of time for completion, time to analyze the data, and patients unable to complete the measures independently as being the top reasons for not using outcome measures. Other limits were related to clinicians not following evidence-based practice guidelines because it is different than the typical work, they do [7, 31]. Several measures can be collected by self-report but are limited to what is asked by the measure. Others like the PSFS are routinely collected by the clinician yet allow the patient to explain their greatest functional limit, pain experience, and reason for searching out physical therapy. Based on the results of this data analysis, further study is needed to determine if there are similar top-ranking categories for the PSFS for other chronic pain conditions. The NDI and the PSFS show only a moderate correlation suggesting each measure has unique aspects but neither alone fully captures what drives the patient to physical therapy. Development of modifications of current outcome measures may encourage higher usage, improve reimbursement, and help practitioners develop a therapy plan that includes the patient-generated concerns to capture the patient's functional limitations and pain experience.

## Figures and Tables

**Table 1:** Neck pain ICD-10 codes with corresponding definitions that were used for inclusion in the study. Screening was done on electronic medical records between January 2013–September 2018.

**ICD-10 code**	**Definition**
M54.2	Neck pain and chronic
M50.90	Cervical pain and disk
M54.12	Cervical pain and radiculopathy
S13.4XXD	Whiplash disorder
R51	Bilateral headache
G44.229	Chronic tension headache
F45.41	Stress headache

**Table 2:** Reported number of activity limitations for each category listed on the Patient Specific Functional Scale (PSFS) for all patients.

**Functional activity limitation**	**Total (2609)**	**Males (928)**	**Females (1681)**
	**Count (%)**	**Count (%)**	**Count (%)**
Movement	496 (19.0%)	213 (22.9%)	283 (16.7%)
Exercise	339 (13.0%)	125 (13.4%)	214 (12.8%)
Sleeping	325 (12.5%)	113 (12.1%)	212 (12.6%)
Sitting/reading/studying	337 (12.9%)	107 (11.5%)	232 (13.8%)
Lifting	216 (8.3%)	73 (7.9%)	143 (8.5%)
Driving	167 (6.4%)	56 (6.0%)	111 (6.5%)
Working	166 (6.4%)	51 (5.5%)	115 (6.8%)
ADLs	129 (4.9%)	44 (4.7%)	85 (5.0%)
Reaching	93 (3.6%)	33 (3.6%)	60 (3.6%)
IADLs	111 (4.2%)	22 (2.4%)	89 (5.2%)
Standing	70 (2.7%)	22 (2.4%)	48 (2.8%)
Computer	68 (2.6%)	21 (2.3%)	47 (2.8%)
Hobbies	51 (2.0%)	30 (3.2%)	21 (1.3%)
Gardening	25 (1.0%)	15 (1.6%)	10 (0.6%)
Lying down	16 (0.6%)	3 (0.3%)	13 (0.8%)

Abbreviations: ADLs, activities of daily living; IADLs, instrumental activities of daily living.

## Data Availability

Original underlying data from the results of this study is not open to the public as per Human Subjects Protection and HIPPA from the University of Iowa. Requests, if needed, can be made to the corresponding author for requisitions of approval to the IRB at the University of Iowa.
