# Supplementary material for: Categories of the Patient-Specific Functional Scale Activities in Chronic Neck Pain and Their Relationship to the Neck Disability Index
Source: Rehabil Res Pract. 2024 Sep 23;2024:3126892. doi: 10.1155/2024/3126892 (PMC11442039; doi:10.1155/2024/3126892)
Supplement: Supporting Information — Additional supporting information can be found online in the Supporting Information section. [file 3126892.f1.docx]

**Appendix 1. PATIENT SPECIFIC FUNCTIONAL SCALE (therapist script at UIHC clinics)**

**Please identify up to 3 important activities** that you are unable to do or are having difficulty with because of your injury or problem.

Please rate your ability to perform each activity today using a "0" to "10" scale, **where "0" means you are "unable to perform" the activity and "10" means you are "able to perform" the activity at the same level as before your injury or problem.**

**Patient-specific activity scoring scheme (point to one number per activity)**

**0 1 2 3 4 5 6 7 8 9 10**

Unable to perform the activity.

Able to perform at the same ability prior to injury or problem.

| **Activity** | **Initial score** |  |  |  |  |
| --- | --- | --- | --- | --- | --- |
| **1.** |  |  |  |  |  |
| **2.** |  |  |  |  |  |
| **3.** |  |  |  |  |  |

Total score = sum of the activity scores/number of activities

Minimum detectable change (90%CI) for average score = 2 points

Minimum detectable change (90%CI) for single activity score = 3 points

Referenced for outline (Stratford et al., 1995)

**Appendix 2. Neck Disability Index**

**Name: Date:**

**Neck Disability Index**

This questionnaire is designed to help us better understand how your neck pain affects your ability to manage everyday life activities. Please mark in each section the one box that applies to you, although you may consider that two of the statements in any one section relate to you. Please mark the box that most closely describes your present situation.

Pai**n Intensity**

- I have no pain at the moment.
- The pain is very mild at the moment.
- The pain is moderate at the moment.
- The pain is fairly severe at the moment.
- The pain is very severe at the moment.
- The pain is the worst imaginable at the moment.

**Personal Care (e.g. Washing, Dressing)**

- I can look after myself normally without causing extra pain.
- I can take care of myself normally, but it causes extra pain.
- It is painful to look after myself and I am slow and careful.
- I need some help but manage most of my personal care.
- I need help every day in most aspects of self-care.
- I do not get dressed. I wash with difficulty and stay in bed.

**Lifting**

- I can lift heavy weights without causing extra neck pain.
- I can lift heavy weights, but it gives me extra neck pain.
- Neck pain prevents me from lifting heavy weights off the floor, but I can manage if items are conveniently positioned, i.e. on a table.
- Neck pain prevents me from lifting heavy weights, but I can manage light weights if they are conveniently positioned.
- I can lift only very light weights.
- I cannot lift or carry anything at all.

**Work**

- I can do as much work as I like.
- I can only do my usual work, but no more.
- I can do most of my usual work, but no more.
- I can’t do my usual work.
- I can hardly do any work at all.
- I can’t do any work at all.

**Headaches**

- I have no headaches at all.
- I have slight headaches that come infrequently.
- I have moderate headaches that come infrequently.
- I have moderate headaches that come frequently.
- I have severe headaches that come frequently.
- I have headaches almost all the time.

**Concentration**

- - I can concentrate fully without difficulty.
  - I can concentrate fully with slight difficulty.
  - I have a fair degree of difficulty concentrating.
  - I have a lot of difficulty concentrating.
  - I have a great deal of difficulty concentrating.
  - I can’t concentrate at all.

**Sleeping**

- - I have no trouble sleeping.
  - My sleep is slightly disturbed for less than 1 hour.
  - My sleep is mildly disturbed for up to 1-2 hours.
  - My sleep is moderately disturbed for up to 2-3 hours.
  - My sleep is greatly disturbed for up to 3-5 hours.
  - My sleep is completely disturbed for up to 5-7 hours.

**Driving**

- I can drive my car without neck pain.
- I can drive my car with only slight neck pain.
- I can drive as long as I want with moderate neck pain.
- I can’t drive as long as I want because of moderate neck pain.
- I can hardly drive at all because of severe neck pain.
- I can’t drive my car at all because of neck pain.

**Reading**

- - I can read as much as I want with no neck pain.
  - I can read as much as I want with slight neck pain.
  - I can read as much as I want with moderate neck pain.
  - I can’t read as much as I want because of moderate neck pain.
  - I can’t read as much as I want because of severe neck pain.
  - I can’t read at all.

**Recreation**

- - I have no neck pain during all recreational activities.
  - I have some neck pain with a few recreational activities.
  - I have some neck pain with all recreational activities.
  - I have neck pain with most recreational activities.
  - I can hardly do recreational activities due to neck pain.
- I can’t do any recreational activities due to neck pain.

**Scoring: ____/50 Transform to a percentage score x 100 = _______% points**

**____________________________________________________________________________________________**

**Scoring:** For each section the total possible score is 5; if the first statement is marked the section score = 0, if the last statement is marked it = 5.

If all 10 sections are completed the score is calculated as follows:

**Example**: 16 (total scored) 50 (total possible scores) x 100 = 32%

If one section is missed or not applicable the score is calculated as follows:

**Example**: 16 (total scored) 45 (total possible score) x 100 = 35.5%

**Minimal Detectable Change**: ( 90% confidence) 5 points or 10% points

**Appendix 3. Flow chart of Methods Process**

Data of 2609 PSFS functional activity limits reviewed and categorized from n = 2283.

Category totals of the most frequent functional activity limits of the PSFS from 2283 individuals with chronic neck pain established.

Data of 1651 PSFS functional activity limits (33%) removed due to error in entry.

15 categories established from review of preliminary data by licensed PT’s and PT students in their final year to use for categorizing PSFS functional activity limits.

Functional activity limits of the PSFS from 3-month preliminary data sorted by lead physical therapist (PT) and reviewed by the 3 other licensed PTs into 17 primary categories.

Categorical differences from 2 independent PT students (333 activities) reviewed by 3^rd^ independent reviewer for reconciliation.

2 independent PT student reviewers sorted functional activity limits PSFS data (2609 activities) into 15 categories.
